# Visual content and thematic analyses of images shared on social media before and after episodes of self-harm in a UK clinical youth sample

**DOI:** 10.1136/bmjopen-2025-103456

**Published:** 2026-01-19

**Authors:** Amanda Bye, Kylee Trevillion, Emma Wilson-Lemoine, Daniel Leightley, Ben Carter, Maria Liakata, Jaycee Hopper, Rina Dutta

**Affiliations:** 1 Department of Child and Adolescent Psychiatry, King’s College London Institute of Psychiatry Psychology & Neuroscience, London, UK; 2 Department of Psychological Medicine, King’s College London Institute of Psychiatry Psychology & Neuroscience, London, UK; 3 Health Service and Population Research Department, King’s College London Institute of Psychiatry Psychology & Neuroscience, London, UK; 4 Department of Population Health Sciences, School of Life Course & Population Sciences, King’s College London, London, UK; 5 Department of Biostatistics and Health Informatics, King’s College London Institute of Psychiatry Psychology & Neuroscience, London, UK; 6 School of Electronic Engineering & Computer Science, Queen Mary University of London, London, UK; 7 The Alan Turing Institute, London, UK; 8 University of Warwick, Coventry, UK; 9 YoungMinds, London, UK; 10 South London and Maudsley NHS Foundation Trust, London, UK

**Keywords:** Suicide & self-harm, Child & adolescent psychiatry, MENTAL HEALTH, QUALITATIVE RESEARCH, Social Media

## Abstract

**Objectives:**

Little is known about how young people use social media during periods of self-harm. This study aimed to explore how they express themselves online through images posted on social media before and after self-harm and how this expression may change across these periods, employing visual content and thematic analyses.

**Design:**

A prospective cohort study, with qualitative analysis conducted using a recurrent cross-sectional approach and codebook methodology, accounting for chronological changes across time points before, during and after episodes of self-harm.

**Setting:**

Participants were recruited from a mental health NHS Trust in the UK.

**Participants:**

Image data during episodes of self-harm was available for 20 participants. The majority of whom were aged 18 years or older (n=15), female (n=14) and met criteria for moderate or severe anxiety and depression (n=18). The sample reflected diverse ethnic backgrounds, with six participants identifying as Asian or Mixed/Multiple ethnic backgrounds.

**Results:**

None of the images investigated had direct visual presentations of self-harm. A few images referenced self-harm through the medium of text, and this was largely to normalise and promote help-seeking. Several themes were identified, including participation in activities that support well-being, love and relationships, connecting through humour, expressions of distress, and promoting mental health awareness and support. Subtle temporal changes were also observed.

**Conclusions:**

Findings suggest that young people may temporarily withdraw from social media on the day of a self-harm event and rarely post graphic self-harm images around that time. This may reflect concerns about being stigmatised, but also improved platform moderation. Instead, platforms may serve as spaces for expressing self-care behaviours and connecting with others about both positive and challenging emotions, and across a range of topics including mental health.

**Trial registration number:**

ClinicalTrials.gov: NCT04601220.

**STRENGTHS AND LIMITATIONS OF THIS STUDY**

- This study demonstrates the feasibility of linking participant-level data with their social media content to enable accurate identification and analysis of images shared online by a known clinical sample of young people during periods of self-harm.
- Co-production was integral to the study from the outset, with young people meaningfully involved across all key stages of the research process to ensure relevance and acceptability for the intended population.
- Although the platforms included in this study are widely used by young people, their usage may not be representative of other platforms that may offer greater anonymity and looser moderation policies.
- Participants’ ability to choose what social media data to share with the study—an essential aspect of ethical research—combined with the burden of providing this data due to platform-imposed restrictions, affected data availability.

## Introduction

 Defined by the UK National Institute for Health and Care Excellence (NICE) as ‘intentional self-poisoning or injury irrespective of the apparent purpose’,^1^ self-harm is highly complex and a strong risk factor for suicide.^2 3^ Its rising prevalence among young people, especially in those with mental health difficulties, is a major public health priority.^3 4^ There is widespread concern that social media may be contributing to these rising rates, particularly through exposure to harmful content and cyberbullying.^5 6^ Two recent systematic reviews found harmful effects of both sharing and viewing self-harm-related images online, suggesting it can reinforce and encourage self-harm behaviours.^7 8^ However, important positive effects of using social media have also been reported, such as its role in mitigating self-harm, supporting recovery and providing opportunities for online peer support, which may be particularly beneficial for marginalised groups.^5 7 8^

Real-world social media data offers unique opportunities to investigate the effects of social media by capturing naturally occurring online behaviours. This approach alleviates the key limitations of self-report, on which much of the available evidence is based.^6^ In this emerging area, studies have relied largely on publicly available data, with qualitative methods employed to provide in-depth, interpretative insights into online behaviour that move beyond surface-level metrics, such as underlying meanings, motivations and social dynamics.^9^ Online ethnography is one such qualitative approach, used for example to explore textual comments on YouTube videos shared by individuals self-identifying with a severe mental illness,^10^ as well as textual and image postings about self-harm on Instagram, Reddit and X.^11^ In the latter study, the authors reported that users may interact with others around self-harm-related content to seek peer support.^11^ They also suggested that users may share graphic self-harm images to convey distress that they find difficult to express in words. Other notable approaches include visual content and thematic analyses in studies investigating self-harm^12^ and disordered eating.^13^ Despite the concerns, Shanahan and colleagues^12^ found little evidence that images shared on Instagram, Tumblr and X explicitly represented self-harm or encouraged self-harm behaviour. Instead, the authors reported that platforms provide a virtual space for users to express their emotions creatively and offer inspiration to others with shared experiences.

Previous studies demonstrate the value of qualitative methods for examining both textual and image data. This is increasingly important given the rise of image-based online communication, particularly through platforms such as Instagram, and allows us to better understand the diversity of self-harm-related social media use. However, existing research has notable limitations, including a lack of linked participant data—most have sampled unknown groups based on publicly available content—and the absence of information on the timing of self-harm events in relation to social media use. As a result, little is known about the individual posting or the broader context beyond what is presented in the content itself, which limits the applicability of the findings at an individual level.

To address these evidence gaps, the Social Media, Smartphone use and Self-harm in Young People (3S-YP) study is the first of its kind to integrate real world social media with temporal information on self-harm events over a 6-month period in a clinical youth sample.^14^ Using this innovative data-linkage cohort, we aimed to examine how young people express themselves online through images posted on social media during periods of self-harm and how this expression may change across these periods, employing visual content and thematic analyses. This exploratory research will assess the feasibility of this novel data-linkage in qualitative research and provide insights into online self-expression during periods of self-harm, with the potential to identify subtle changes that may signal early risk indicators. Such findings could help inform approaches to big data analytics,^15^ as well as the development of prevention and intervention strategies.

## Method

### Study design

This study employed a prospective cohort design linking real-world social media data with questionnaire and electronic health records (EHR) data. Qualitative analysis followed a recurrent cross-sectional approach^16^ using a codebook methodology,^17^ accounting for chronological changes in the visual content and thematic representations of images across three key time points during episodes of self-harm (ie, the 7 days before an event, the day of the event itself and the 7 days following—herein termed the ‘event window’). Full details on the 3S-YP study methods are available elsewhere.^14 18^ We have reported this analysis in accordance with the COnsolidated criteria for REporting Qualitative research (COREQ) guidelines (see online supplemental file 1).

### Patient and public involvement

We worked with Senior Service User Consultant and Co-Investigator, Stella Branthonne-Foster, and leading UK youth mental health charity, YoungMinds, to actively involve young people with lived experience in this research from the outset, enhancing relevance and acceptability. This included consultations, focus groups, workshops and participation in the 3S-YP Steering Group. Youth advisors were involved in formulating the research questions, designing and overseeing the study, considering ethical issues, developing digital tools and participation information materials, interpreting findings and contributing to dissemination plans, including co-authorship (JH).

### Research team

The 3S-YP team is multidisciplinary, including Epidemiology (EW-L, RD), Health Sciences (AB, KT), Natural Language Processing (ML), Psychiatry (RD), Psychology (AB, KT, EW-L), Software Engineering (DL) and Statistics (BC). Those involved in the analysis are experienced qualitative and participatory researchers (AB, KT, EW-L, RD), with representation from service user (EW-L) and youth advisory perspectives (JH). The analysis was led by AB, with support from EW-L and KT, and clinical oversight from RD. Analysts and RD had regular meetings, with interpretative input from JH, to ensure the diverse disciplinary and lived experience perspectives informed every stage. The analysis took a critical realist approach, assuming the images reflect young people’s subjective and observable online presentations rather than objective reality.^19^

### Recruitment and data collection

Young people aged 13–25 years were recruited between June 2021 and November 2022 from the UK’s largest mental health service provider, South London and Maudsley NHS Foundation Trust, via a patient research participation register and followed up for 6 months. Young people were invited to participate via text message, with a follow-up after 1 week. They accessed the study information and provided digital consent (or assent) through a secure online enrolment system via a unique weblink. Questionnaire data were collected at baseline and monthly intervals during follow-up. At baseline and months 3 and 6, participants uploaded a copy of their data from Facebook, Instagram, Snapchat, TikTok, X and YouTube to the secure system via a unique link. EHR data were extracted after the baseline questionnaire and on study completion. All study data were assigned a unique identifier and stored securely on restricted-access servers in compliance with the UK General Data Protection Regulation and the Data Protection Act (2018). Social media data were stored separately from identifiable data. Data handling adhered to robust study protocols to ensure confidentiality.

### Measures

#### Self-harm

Self-harm events during follow-up were identified from self-report and clinician-recorded data. A questionnaire version of the Child and Adolescent Self-harm in Europe (CASE) study criteria^20^ was administered once a month. Information on clinician-recorded events was extracted from free-text and structured fields in the EHR after study completion. Events were excluded if the timing could not be determined. We used a broad definition to classify self-harm behaviours, consistent with NICE guidelines^1^ and previous research on clinically recognised types^1 21 22^ while also including behaviours not traditionally recognised as self-harm when there was a stated intention to self-harm. This comprised (1) self-poisoning: ingestion of non-recreational drugs above the recommended dose, poisonous amounts of recreational drugs within a discrete event or substances not intended for human consumption, with intent to self-harm; (2) self-injury: intentional self-inflicted injuries (eg, cutting, biting, burning), excluding habitual or repetitive self-injurious behaviours; (3) both self-poisoning and self-injury: events involving both types of behaviour; and (4) other types of self-harm: for example, attempted strangulation or drowning—regardless of whether injury was sustained or whether these behaviours occurred in isolation or alongside self-poisoning or self-injury—as well as alcohol poisoning, prolonged substance misuse or disordered eating behaviours, provided these latter behaviours were accompanied by a stated intention to self-harm.

#### Social media data

All images posted by participants during an event window were extracted from their most recent valid data upload. Images posted by a user are available in their data files provided by the social media platforms, but images in which the user has been tagged by others are not. The image data included a broad range of formats, such as photographs, pictures, textual images (ie, words only) or a combination of these within a single image. Images could be posted with textual captions that appear alongside or, in the case of Instagram stories, overlaying them. Instagram stories also offer other creative features, such as comment boxes or polls, that similarly overlay the image. In line with participant consent, no data shared through private messages were processed. No image data were available from Snapchat, TikTok or YouTube for any participants.

#### Other measures

We collected baseline socio-demographics and symptoms of depression, anxiety and sleep disturbance, assessed using the Patient Health Questionnaire-9,^23^ Generalised Anxiety Disorder Scale -7^24^ and Paediatric Sleep Disturbance Short Form V1.0 4a (for participants under 18 years of age)^25^/Patient-Reported Outcomes Measurement Information System Sleep Disturbance Short Form V1.0 4a (for participants aged 18 years or older),^26^ respectively. Primary mental health diagnosis and current service user status were extracted from the EHR following study participation.

### Sampling strategy

Of the total 3S-YP sample (n=362), one-third (n=122) had at least one self-harm episode during the 6-month follow-up. Among these, 25 participants posted on social media during an event window, and 20 had at least one image available for this analysis. For participants with multiple self-harm events, we used the first event with image data. If an image was posted more than once, we only included the first such image (resulting in exclusion of 40 duplicates). Where a participant posted three or more different images within the same day, time slot and modality, two were randomly selected (resulting in a total of seven excluded images). These a priori decisions to remove duplicates and limit the number of images per participant aimed to improve efficiency and reduce potential data bias, while retaining the metadata log for reference.

### Analysis

Following a literature review of related evidence, KT developed the initial analysis plan and visual content and thematic analysis codebook, in collaboration with AB and RD (adapting published analytical approaches, including Lavis & Winter, 2020; Mazur & Li, 2016; Pila et al., 2017; Seko & Lewis, 2016; Shanahan et al., 2019).^11^,^1327 28^ The Cry of Pain theory^29 30^ was also applied for images representing self-harm as part of the broader thematic analysis, including coding indicators of perceived stressors, perceived ability to escape, and potential rescue. The analysis plan and coding frames were refined by AB and KT after piloting with ‘test’ cases and finalised by AB during the initial stages of data coding (see online supplemental file 2). Images were coded by EW-L, with training and supervision provided by AB. Each image was coded, initially independently of any other content and then considering any accompanying captions and other overlaying content. A random 20% of images were independently double coded by AB and KT. Rather than achieving consensus, dual coding provided greater depth and encouraged a reflexive approach that encompassed the varied disciplinary perspectives.^31^ Coding was reviewed by AB before undertaking the final stages of the analysis. The latter stages of the visual content analysis were conducted in Excel, where codes were reviewed and categorised. Image counts were prepared to show how many images displayed each visual feature. The remaining stages of the thematic analysis as described by Braun and Clarke^32^ were conducted in NVIVO: (3) generate initial themes, (4) review and develop themes, (5) refine, define and name themes and (6) report writing. Theme generation was iterative, with continual reference to the data to validate and refine the themes. The finalised themes were applied to each time point within the event window in chronological order. Data from all participants were analysed as a unit, enabling comparison across different time points. Analysts documented their ideas to complement the analytical process and met regularly to discuss the developing analysis.

## Results

### Sample descriptives

Of the 20 participants with social media images during an event window, most were aged 18 years or older (n=15), female (n=14) and in some form of education (n=13) (see online supplemental file 3). The sample reflected diverse ethnic backgrounds, with six participants identifying as Asian or Mixed/Multiple ethnic backgrounds. The majority were active in secondary mental health services (n=11) and met criteria for moderate or severe anxiety and depression (n=18). Across the sample, the total number of self-harm events ranged between 1 and 18, with a median of 3 (2, 5). Of the events included in the analysis, self-injury was the most common method.

### Image posting patterns

There were 99 social media images available from the 20 participants. Most images had been posted on Instagram (n=91), with far fewer on Facebook (n=6) or X (n=2). Images were typically posted before or after the self-harm event (n=46; n=47, respectively), rather than on the day itself (n=6). Before and after the self-harm event, images were more often posted between 12 pm and 6 pm (n=23; n=22, respectively), whereas on the day of the event, images were more often posted between 6 am and 12 pm (n=3). The number of images posted per participant across the three time points ranged between 1 and 25, with a median of 2 (1, 10).

### Visual content findings

Most images were photographs (n=50), while others were text-only, such as short written prose on a plain background (n=13), or combined photography and/or drawings with text (n=36) (see online supplemental file 4). These composite images were typically computer-generated, with the text aiding interpretation. The main subject was often a person or group of people (n=39)—typically females—or text (n=28), and this was generally consistent across time points. A range of visual tones and colours were represented, though mid-tones (n=49) and black, grey or white (n=34) were most common. Across time points, a greater proportion of images posted on the day of an event were photographs (n=4) and used dark tones (n=2).

No visual presentations of self-harm were identified in any of the images. A small number referenced self-harm or suicidality in the textual content (n=4), none of which included photographs. These were largely about normalising self-harm and encouraging help-seeking, for example, a cartoon strip illustrating the protective function of peer support. One image explicitly rejected sharing visual presentations of self-harm on social media. None of the images included content that could be interpreted as endorsing or idealising self-harm.

Images portrayed a broad spectrum of emotions, ranging from positive (n=54) to negative (n=26) (eg, frustration, sadness). The emotional tone in some images was difficult to interpret (n=19), including those reflecting conflicting emotions. Many appeared to be used for sharing personal feelings (n=35)—interestingly, none on the day of an event—or daily activities (n=33). A smaller number contained humorous content (n=9), and others offered support, such as validating mental health experiences or sharing resources (n=5).

Around half of the images had an overlaying caption (n=44) or other creative content (n=9), while very few had captions alongside the image (n=7). Among the images with captions, 16 included emojis, most commonly a smiley face or mending heart, but none contained hashtags.

### Thematic representations

We identified eight themes in the image data and present these alongside a separate section detailing the temporal changes observed (see online supplemental file 5).

### Participation in activities that support well-being

This first theme describes images depicting activities that promote well-being. This included creative outlets and pleasurable and health-focused activities, which were typically represented in photographs accompanied by captions to offer context and convey the emotional significance. Images representing creative content creation were predominantly about making music. For example, an image of a young person playing an electric guitar, with the focus on their hand strumming the guitar strings and a caption expressing their passion for playing the instrument. Examples of pleasurable activities included photographs of travelling and outings, some of which featured a small group of people, interpreted as family members or friends. Other images within this theme were health-focused, such as a post-run selfie that had been posted before a self-harm event, with positive emotions attributed to the image through captions.

### Representations of love and relationships

The second theme addressed images portraying love and relationships, and this included diverse forms of social connectedness and loss of love. Images were interpreted as conveying emotions rather than seeking engagement or evoking a response from others and tended to be personal in nature. Social connectedness to others, including family, friends and pets, was often documented and celebrated through photographs, while textual descriptions were less common. Images predominantly depicted in-person social connections, such as group photographs showing positive and reciprocal facial expressions and body language. Examples of online peer support were also shared through screenshots of online community discussions. Loss of love was conveyed through photographs with darker tones. For example, an image posted after a self-harm event was a photograph of several family members posing together presented on a plain black background, accompanied by a caption detailing a family bereavement and echoing the sentiment conveyed in the image. Another aspect of loss—unrequited love—was reflected through textual images, for instance, a short illustrative poem.

### Connecting through humour

The third theme describes images that presented humorous content, including light-hearted content, humour-based depictions of mental health-related content, satire and private jokes. This theme was reflected in images with some textual content and rarely featured people. Images representing light-hearted and trivial matters were often accompanied by captions conveying humour where the meaning was not apparent from the visual alone. For example, a photograph posted following self-harm about a new beverage being marketed, with captions inviting others to comment on its appeal. There were several images that used humour to depict mental health-related content, some of which were interpreted as being used to detract from the underlying emotion. Examples include an image posted before an event that used a comedic film reference to illustrate they were experiencing a personal crisis, and a textual image posted after an event about masking mental health difficulties, with the suggestion the individual deserves an ‘oscar’ for managing a recent crisis, alongside a positive reference to gender diversity. Another image presented a derogatory portrayal of mental health, featuring a Freudian-style therapy session with a text description about visual hallucinations, parodying the irrationality of the experience. A few images presented satirical cartoon illustrations, such as one aimed at pro-life male supporters. This theme also included images reflecting the sharing of in-jokes between close friends, accompanied by captions that included emojis emphasising the individual’s reaction to the shared content.

### Expressions of distress

The fourth theme describes images that conveyed distress. Moderate to high levels of distress were typically conveyed through textual images. A range of emotions were portrayed, including anxiety, low mood, hopelessness, interpersonal distress and social withdrawal. For example, a description about a recent mental health crisis on a plain black background posted before a self-harm event, with a caption indicating it was intended as an update for their friends. Another image posted on the day of an event, similarly with text on a plain background, stated that photographs of self-harm wounds *“*…should not be shared online…*”*, highlighting the distinction between visible scarring and wounds, and sharing online versus in-person. Images within this theme were about communicating distress and not about seeking help. Milder forms of upset or frustration were generally communicated through photographs (or combined photograph and text) and accompanied by captions.

Application of the Cry of Pain theory for the few images referencing self-harm identified one that was interpreted as describing a perceived stressor, that is, the triggering effect of sharing graphic self-harm images online. Regarding the perceived ability to escape and potential rescue factors, a couple of images referenced disclosure and access to support (including signposting and peer support), while another was interpreted as conveying a sense of powerlessness in ‘escaping’ their anguish.

### Promoting mental health awareness and support

The fifth theme describes images promoting mental health awareness and support. Raising awareness was largely expressed through text, either solely textual images or text overlaying photographs or incorporated into drawings. Images addressed issues such as stigma, misperceptions and trivialisation of mental health difficulties. For example, one image posted following an event showed a male actor with a quote overlaying the photograph stating that the harmful portrayal of borderline personality disorder in the media can perpetuate stigmatising attitudes and inhibit disclosure. Images depicting offers of support were all mixed text and photographs or drawings and none were captioned with additional text. Support was explicit, for example, a computer-generated picture posted before a self-harm event of a young female with her head in her lap and an outstretched hand reaching toward her, with the details of support services for self-harm displayed above. There were also more subtle examples, including images offering validation and hopeful imagery, such as a photograph posted following an event of a staircase, possibly symbolic of progress, with a motivational quote on the staircase wall. Several images also referred to receiving support—specifically online and in-person peer support, and these were frequently captioned with text and emojis (eg, mending heart) highlighting the benefits.

### Incongruent atmosphere and tone

The sixth theme cuts across several other themes and describes images where there was an incongruence in the atmosphere and tone. For example, a selfie posted following self-harm of a young person with a neutral or sad facial expression that contrasted with the tone of an applied filter and the caption conveying their enthusiasm. Another example is a photograph with mostly dark tones of a young female with a morose expression and her gaze directed away from the camera, that contrasted with a caption expressing her excitement, again with the use of punctuation for emphasis.

### Diverse expressions of self and sexualisation

The seventh theme describes images with diverse visual representations of the self and sexualisation. This included celebrations of individualism, feminine and masculine sexualisation, blank or neutral facial expressions, and body positivity. This theme was predominantly reflected through photographs, less so drawings, with minimal textual content. Individualism and gender non-conformity were represented in images, for example, a photograph posted before an event of a young Black female artist featuring a natural hairstyle and masculine tailored suit, in front of a purple background. Sexualisation was suggested in images, such as a posed selfie of a young male in underwear displaying a muscular physique and another of a satirical pro-life cartoon featuring a hypersexualised nurse. These types of images differed from other individual portrait photographs of young people with blank or emotionally neutral facial expressions, gazing directly into the camera. A few images were interpreted as promoting body positivity, specifically the female body, such as a selfie posted following a self-harm event of friends posing in swimwear with joyful facial expressions.

### Social activism against injustice

The final theme describes images depicting activism against various forms of social injustice. This included impersonal images with striking and provoking imagery, most likely re-shares of content generated by other users without any accompanying captions. An example of this was a photograph of a war-torn city to encourage support for those affected. Another textual image posted after an event communicated a faith-based ruling regarding same-sex marriage, with a caption that conveyed the personal relevance and support for those affected.

### Temporal thematic changes

Images reflecting wellbeing-related activities were posted both before and after a self-harm event, with no meaningful differences between these time points. On the day of an event, only the sub-theme of pleasurable activities was reflected, and these images lacked accompanying captions. Both sub-themes of love and relationships were present in images posted before and after self-harm, whereas only social connectedness was reflected on the day of an event, typically through photographs of small groups of family or friends. All forms of humorous content were posted before an event, with more posts at this time point than at others. The event day featured only light-hearted content, while posts after the event included both light-hearted and humour-based depictions of mental health. Images posted before and after an event reflected mild to severe distress, whereas few images posted on the day of an event conveyed this theme. All aspects of promoting mental health awareness and support were represented in images posted before and after the event, with comparable proportions across these time points; however, only a single awareness-related image was posted on the day of an event. Incongruence was reflected in images posted before and after the event but absent on the day itself. Within the theme of self and sexualisation, images portraying individualism were only present prior to an event. On the event day, a single image depicting sexualisation was shared, with no other aspects of this theme represented at this timepoint. Activism of an impersonal nature was present prior to self-harm, whereas the single post after the event reflected a personal form of activism, and neither type was posted on the day of an event.

## Discussion

We undertook visual content and thematic analyses of images shared on social media by a clinical sample of young people during periods of self-harm, characterising how they express themselves online and exploring temporal changes. Results reveal a broad spectrum of imagery was posted, the majority of which did not reference self-harm. Instead, a range of themes were reflected from self-care and social support to emotional distress and promoting awareness and support for mental health difficulties. Subtle changes over time were observed, with notably less images posted on social media on the day of self-harm, along with evidence of masking and attempts to downplay distress, such as through humour.

Our findings are consistent with Shanahan *et al*,^12^ who found little evidence of explicit self-harm imagery despite the authors intentionally selecting social media images tagged as self-harm. Although our sample was actively experiencing self-harm during the periods of social media activity, their social media image postings showed no clear signs of deteriorating mental well-being. Instead, the images were diverse in nature, with subtle visual content and thematic changes and indications of a possible temporary digital withdrawal on the day of an event—raising important questions about risk detection and intervention. While much of the current discourse is framed around the harmful effects of sharing and viewing graphic self-harm images online,^7 33^ our results suggest that sharing such images may not be widespread, at least on platforms with greater public visibility. Young people may not share explicit self-harm-related content online due to fear of being stigmatised or concerns about triggering others.^34^ Platforms also continue to improve automated moderation of harmful content,^35^ so the low proportion of self-harm-related images likely reflects a changing digital landscape.^36^ This underscores the relevance of the present study, as future risk detection approaches may need to shift from overtly harmful content to more nuanced or indirect indicators, such as those identified in our findings, using advanced computational image analysis approaches. Among the few images referencing self-harm in the textual content, both personal and socially driven motivations for sharing were reflected.^37^ Content seemed to serve as a means of emotional expression and seeking or offering support—this pattern was also observed in the other mental health-related images. Our application of the Cry of Pain theory identified perceived stressors and potential rescue factors, including signposting and peer support. These findings are in line with previous studies that have examined the underpinning motivations for self-harm.^29 30^ We interpret these findings cautiously, given the absence of linked interview data to clarify participants’ motivations for self-harm and image sharing, which represents an interesting avenue for future research.

Our findings extend the conversation around youth self-harm and social media by highlighting the opportunity for online identity curation and the breadth of interests that co-exist during periods of self-harm^38^—ranging from creative outlets to sports and travel. Sharing images of these types of activities online may serve a self-care function and reflect a desire among young people to be perceived by others as proactive in self-managing their mental health and ‘more than’ their self-harm.^39 40^ Nuanced temporal shifts in this type of content on the day of self-harm may indicate a temporary withdrawal from a young person’s curated online identity. While not necessarily a strong marker for self-harm risk (or mental health deterioration more broadly) on its own, it could form part of a set of weaker, indirect indicators that—combined with other digital activity data—may inform future risk detection and intervention strategies. Other research suggests images such as those observed in our study can play an important role in maintaining offline relationships by enabling young people to share moments with those who are not present at the time a photograph was taken.^41^ Their findings also indicate that sharing humorous content on social media can create opportunities for interaction—a theme strongly reflected in our own results—while inspirational posts can foster deeper, more meaningful connections. Compared with text-based platforms such as X, image-based platforms like Instagram can offer greater intimacy and have been associated with reduced loneliness and increased life satisfaction among young adults.^42^ Fostering a sense of connection through online communication and curating a timeline of these types of positive memories may be particularly valuable for young people experiencing mental health difficulties, who may be more likely to experience social isolation offline.^43 44^

Mental health-related content was prevalent across our dataset. This included text-based descriptions of distress and offers of support and validation.^11 45^ Given the stigma and misperceptions about mental health and self-harm, young people may be reluctant to discuss their difficulties in their offline relationships and so may use social media to communicate with others who share similar experiences.^10 46^ It has been suggested that disclosing mental distress on social media can positively influence well-being through greater perceived connectedness and social support.^47 48^ We found high levels of distress conveyed through text-based images, suggesting potential for early risk detection using automated computational approaches such as text extraction and sentiment analysis. A recent study using natural language processing to predict distress from social media images and captions found that, in line with our findings, users tend to post more greyscale images and fewer images featuring family or peer networks during periods of distress.^49^ Our results also suggest that incongruence between the atmosphere and tone of some images may reflect an attempt to mask mental health difficulties. For example, bright colours with a sombre caption, or a dark background or filter with a cheerful caption. Social media enables selective self-presentation,^50^ and research has found that self-expression inconsistent with one’s ‘true self’ can negatively affect well-being and social connectedness^51 52^—both risk factors for self-harm and suicide, and thus possible targets for intervention.^53 54^ For some young people, incongruent imagery prior to self-harm may signal an opportunity for intervention and warrant further exploration at an individual level.

Representations of self-expression and individualism were also observable in our data. Our findings align with studies of college students,^55 56^ which found Instagram and Snapchat were preferred over Facebook and X as platforms for self-expression. One explanation is that young people may feel freer to express themselves on Instagram compared to Facebook because fewer of their parents’ generation use the platform, and they may feel less constrained by societal expectations.^57 58^ Several images within our study revealed expressions of one’s sexuality, including those posted by gender diverse young people, a group who may be more likely to use social media to express themselves than their heterosexual peers and may particularly benefit from online networks.^59^

### Strengths and limitations

Previous studies selected their data based on content with no information available on the individual posting or the circumstances around the time of posting (eg, Pila *et al*,^13^ 2017; Shanahan *et al*,^12^ 2019), whereas the novel data linkage in this study has enabled us to examine posting behaviour among a known clinical youth sample during periods of self-harm. A wealth of data is available on our sample, and this has enabled the triangulation of self- and clinician-reported self-harm with real-world social media behaviour. The analytic framework generated from this analysis will provide a valuable foundation for addressing key follow-up research questions with this cohort, including how themes map at an individual level and how they compare with non-cases. Furthermore, this study brings together a range of disciplinary perspectives and lived experience.

Although the platforms examined in this analysis are widely used by young people,^60 61^ our findings may not generalise to other platforms, particularly those offering greater anonymity and looser moderation policies.^44^ While participants valued personal agency in choosing what data to share with this study, some may have been reluctant to share their data due to data privacy concerns and so may have omitted certain information.^62^ Furthermore, due to data access restrictions imposed by platforms,^63^ consenting young people had to manually provide their data to the study. This placed a significant burden on participants and required a degree of technical literacy, which may have also impacted on data availability. At the time of publishing our protocol,^14^ the intention was to analyse up to 600 images posted before an event from a purposive sample. Instead, due to data availability, we included all cases with image data available before and after self-harm. While duplicate images were represented thematically, their removal and limits on participant images may have led to missed insights on temporal changes, although the number excluded was small. Finally, we did not analyse other multimedia content, for example, videos, which may offer users more creative ways to conceal self-harm content from moderators and provide further insight into how clinical youth use social media.^64^

## Conclusion

This analysis, which is the first from our study and within the field, explored visual content and thematic representations of images posted on social media by young people during episodes of self-harm. Our findings provide important insights into the diverse ways young people express themselves online and reveal subtle changes over time. The low frequency of self-harm-related imagery likely reflects broader shifts in the digital landscape. Instead, social media appears to serve as a space for self-care, emotional expression and connection with others, around a range of topics, including mental health. Our findings underscore the need for future detection efforts to focus on more nuanced or indirect indicators of self-harm risk and tackle attempts to mask or detract from mental distress. When combined with other digital behavioural data and integrated within a multimodal approach, the image signals identified in this study could inform more effective and valid strategies for risk detection and intervention, rather than functioning as a standalone flag. Future research should examine the relevance of these findings at an individual level and using automated computational approaches in big data to better understand the significance of the subtle changes detected across time points.

## Supplementary material

10.1136/bmjopen-2025-103456 online supplemental file 1

10.1136/bmjopen-2025-103456 online supplemental file 2

10.1136/bmjopen-2025-103456 online supplemental file 3

10.1136/bmjopen-2025-103456 online supplemental file 4

10.1136/bmjopen-2025-103456 online supplemental file 5

## Data Availability

Data are available upon reasonable request.
