# Supplementary material for: Visual content and thematic analyses of images shared on social media before and after episodes of self-harm in a UK clinical youth sample
Source: BMJ Open. 2026 Jan 19;16(1):e103456. doi: 10.1136/bmjopen-2025-103456 (PMC12820819; doi:10.1136/bmjopen-2025-103456)
Supplement: online supplemental file 2 [file bmjopen-16-1-s002.docx]

**Supplementary File 2**

**Visual Content Analysis – Coding frame**

| **Category** | **Description** |
| --- | --- |
| Platform | Facebook, Instagram, Twitter |
| Date of posting | In the seven days before a self-harm event, on the day of a self-harm event, in the seven days following a self-harm event |
| Type of image | Photograph, picture (i.e. drawing, painting, or computer-generated artwork), textual (i.e. embedded text, not including overlay text captions), combination of these or a collage |
| Textual image | Describe any textual content (i.e. embedded text, not including overlay text captions), including emojis, hashtags, language, location |
| Text caption | Describe any text caption accompanying the image (text, emojis, hashtags, language, location [i.e. alongside or overlaying the image]). |
| Additional overlay creative content | Describe any additional creative content overlaying the image (e.g. comment box, location pin, clock or timer, website address, account handle) |
| Intended use of social media site | To interact with friends or family, networking, educational, unclear |
| Photo techniques | Predominant colour, tone, type of lighting (natural/artificial), shading |
| Main subject | Animal, fictional character, individual, group of people, object, landscape |
| Gender (if includes a person) | Male, female, gender other than binary, unclear |
| Age (if includes a person) | Infant, child, teenager, adult, unclear |
| Emotional tone | Positive, negative, mixed, ambiguous |
| Stated purpose of post | E.g. inform others, legitimise, seek support, share recovery, inspire, explain, share feelings, share humorous content, respond to others, ambiguous |
| Stated emotion | Anger, sadness, hope, fear, pride, happiness, unclear |
| Topic | E.g. daily routine, employment, education, relationships, emotions, self-harm, other |
| Description of visual content |  |
| Self-harm representation | Is self-harm represented? |
| Description of self-harm representation | Active self-harm, self-harm injury or scar, severity (e.g. superficial, moderate, severe), body part (if visible), method of self-harm referenced |
| Purpose of self-harm content | Instructional content (e.g. description for others to follow), reward-based (e.g. endorses self-harm as a way of managing distress), idealise, normalise, reject, neutral response to self-harm behaviour |

**Thematic Content Analysis – Coding Frame**

| **Category** | **Description** |
| --- | --- |
| Platform | Facebook, Instagram, Twitter |
| Date of posting | In the seven days before a self-harm event, on the day of a self-harm event, in the seven days following a self-harm event |
| Type of image | Photograph, picture (i.e. drawing, painting, or computer-generated artwork), textual (i.e. embedded text, not including overlay text captions), combination of these or a collage |
| Textual image | Describe any textual content (i.e. embedded text, not including overlay text captions), including emojis, hashtags, language, location |
| Text caption | Describe any text caption that accompanies the image, including emojis, hashtags, language, location (e.g. alongside or overlays image) |
| Description of visual content | Describe the setting and characters (e.g. appearance, dress, facial expression, sex, gender, age, relationship, social status, nationality, religion, culture) |
| Facial and behavioural expression | Describe the emotions conveyed through the characters’ facial and behavioural expressions |
| Description of textual content | Describe any textual content (i.e. embedded text, not including overlay text captions), including emojis, hashtags, language, location |
| Image background | Describe the background (e.g. colour, type of lighting [natural/artificial], shading |
| Image foreground | Describe the foreground (e.g. colour, type of lighting [natural/artificial], shading) |
| Central elements | Describe the elements that are centred in the image |
| Atmosphere | E.g. happy, positive, moody, dark |
| Emotions | Describe the emotions the image evokes |
| Function | Describe what particularly captures your attention |
| Interpretation including text caption | Read the text caption. Does it provide any new information? Describe your new interpretation of the image |
| Symbolism/visual rhetoric | Describe any symbolism and/or visual rhetoric in the image |
| Intertextuality | Does the image remind you of another image (e.g. artwork)? If so, describe |
| Overriding impression | Describe your overriding impression from the image |
| Cry of Pain theory | Presence of Cry of Pain theory factors – perceived stressors (e.g. indicators of defeat, rejection, loss), perceived ability to escape (i.e. escapable – not escapable), potential rescue factors (e.g. availability of social support, positive future thinking) |
