# Supplementary material for: Visual content and thematic analyses of images shared on social media before and after episodes of self-harm in a UK clinical youth sample
Source: BMJ Open. 2026 Jan 19;16(1):e103456. doi: 10.1136/bmjopen-2025-103456 (PMC12820819; doi:10.1136/bmjopen-2025-103456)
Supplement: online supplemental file 3 [file bmjopen-16-1-s003.docx]

**Supplementary File 3: Sample characteristics**

| Characteristics^a^ | Participants  (n=20) |  |  |
| --- | --- | --- | --- |
|  | n (%) | Mean (SD) | Median (IQR) |
| Age in years |  | 19 (3) | 19 (18, 20) |
| Age categories |  |  |  |
| 13 – 17 years old | 5 (25%) |  |  |
| 18 – 25+ years old | 15 (75%) |  |  |
| Gender |  |  |  |
| Female | 14 (70%) |  |  |
| Male | 2 (10%) |  |  |
| Prefer to self-describe | 4 (20%) |  |  |
| Ethnicity |  |  |  |
| Any Asian or British Asian background | 3 (15%) |  |  |
| Any Mixed or Multiple ethnic background | 3 (15%) |  |  |
| Any White background | 14 (70%) |  |  |
| Education status |  |  |  |
| Secondary school/college/university/other | 13 (65%) |  |  |
| None of the above | 7 (35%) |  |  |
| Employment status^b^ |  |  |  |
| Employed/self-employed | 10 (50%) |  |  |
| Student | 13 (65%) |  |  |
| Not working for health reasons/unemployed | 4 (20%) |  |  |
| Highest level of education |  |  |  |
| No formal qualifications | 1 (5%) |  |  |
| GCSE grades 9-4/A*-C, NVQ level 2 or  equivalent | 6 (30%) |  |  |
| AS/A Level or above | 12 (60%) |  |  |
| Missing | 1 (5%) |  |  |
| Current mental health service user status |  |  |  |
| Active | 11 (55%) |  |  |
| Discharged | 9 (45%) |  |  |
| Primary mental health diagnosis |  |  |  |
| Mood (affective) disorder (F30-39) | 3 (15%) |  |  |
| Anxiety, dissociative, stress-related, somatoform  and other nonpsychotic mental disorders (F40-48) | 3 (15%) |  |  |
| Behavioral syndromes associated with  physiological disturbances and physical factors  (F50-59) | 4 (20%) |  |  |
| Disorders of adult personality and  behavior (F60-69) | 3 (15%) |  |  |
| Pervasive and specific  developmental disorders (F84) | 2 (10%) |  |  |
| Behavioral and emotional disorders with onset  usually occurring in childhood and adolescence  (F90-98) | 2 (10%) |  |  |
| Other | 2 (10%) |  |  |
| Missing | 1 (5%) |  |  |
| Generalised Anxiety Disorder (GAD-7) | | 15 (5) |  |
| Minimal/mild anxiety | 2 (10%) |  |  |
| Moderate/severe anxiety | 18 (90%) |  |  |
| Patient Health Questionnaire (PHQ-9) | | 18 (6) |  |
| Minimal/mild depression | 2 (10%) |  |  |
| Moderate/moderately severe/severe depression | 18 (90%) |  |  |
| PROMIS Pediatric Sleep Disturbance Short Form (n=5) |  | 62 (6) |  |
| Within normal limits/mild sleep disturbance | 2 (40%) |  |  |
| Moderate/severe sleep disturbance | 3 (60%) |  |  |
| PROMIS Sleep Disturbance Short Form (n=15) |  | 59 (6) |  |
| Within normal limits/mild sleep disturbance | 9 (60%) |  |  |
| Moderate/severe sleep disturbance | 5 (40%) |  |  |
| Number of self-harm events during 6-month follow-up |  | 5 (4) | 3 (2, 5) |
| 1 | 3 (15%) |  |  |
| 2 - 5 | 12 (60%) |  |  |
| ≥6 | 5 (25%) |  |  |
| Method of self-harm^c^ |  |  |  |
| Self-poisoning | - |  |  |
| Self-injury | 13 (65%) |  |  |
| Both self-poisoning and self-injury | 1 (5%) |  |  |
| Other types of self-harm | 1 (5%) |  |  |
| Missing | 5 (25%) |  |  |

^a^Missing only included as a separate row for variables with missing data.

^b^Young people were able to select all response options that were relevant.

^c^Method of self-harm for the events included in the analysis.
