# Supplementary material for: Visual content and thematic analyses of images shared on social media before and after episodes of self-harm in a UK clinical youth sample
Source: BMJ Open. 2026 Jan 19;16(1):e103456. doi: 10.1136/bmjopen-2025-103456 (PMC12820819; doi:10.1136/bmjopen-2025-103456)
Supplement: online supplemental file 4 [file bmjopen-16-1-s004.docx]

**Supplementary File 4: Visual content findings**

| **Visual content** | **Total images**  **(n = 99)** | **Before**  **(n = 46)** | **On the day**  **(n = 6)** | **After**  **(n = 47)** |
| --- | --- | --- | --- | --- |
|  | **n (%)** | **n (%)** | **n (%)** | **n (%)** |
| Image type |  |  |  |  |
| Photographs | 50 (51%) | 22 (48%) | 4 (67%) | 24 (51%) |
| Picture | - | - | - | - |
| Textual | 13 (13%) | 6 (13%) | 1 (17%) | 6 (13%) |
| Combination | 36 (36%) | 18 (39%) | 1 (17%) | 17 (36%) |
| Predominant visual tone |  |  |  |  |
| Light tones | 32 (32%) | 15 (33%) | 2 (33%) | 15 (32%) |
| Mid tones | 49 (50%) | 23 (50%) | 2 (33%) | 24 (51%) |
| Dark tones | 18 (18%) | 8 (17%) | 2 (33%) | 8 (17%) |
| Predominant colour |  |  |  |  |
| Beige/brown | 13 (13%) | 5 (11%) | 2 (33%) | 6 (13%) |
| Blue | 17 (17%) | 5 (11%) | 1 (17%) | 11 (23%) |
| Green | 8 (8%) | 1 (2%) | - | 7 (15%) |
| Black/grey/white | 34 (34%) | 19 (41%) | - | 15 (32%) |
| Orange/red/yellow | 11 (11%) | 8 (17%) | 1 (17%) | 2 (4%) |
| Pink/purple | 16 (16%) | 8 (17%) | 2 (33%) | 6 (13%) |
| Main subject |  |  |  |  |
| Animal | 7 (7%) | 4 (9%) | - | 3 (6%) |
| Fictional character | 7 (7%) | 5 (11%) | - | 2 (4%) |
| Landscape | 5 (5%) | - | - | 5 (11%) |
| Object | 13 (13%) | 4 (9%) | 1 (17%) | 8 (17%) |
| Person/group of people | 39 (39%) | 20 (44%) | 4 (67%) | 15 (32%) |
| Text | 28 (28%) | 13 (28%) | 1 (17%) | 14 (30%) |
| Gender of individual/group (n = 39) |  |  |  |  |
| Female | 21 (54%) | 12 (60%) | - | 9 (60%) |
| Male | 3 (8%) | 1 (5%) | - | 2 (13%) |
| Mixed gender group | 9 (23%) | 3 (15%) | 4 (100%) | 2 (13%) |
| Other/unclear | 6 (15%) | 4 (20%) | - | 2 (13%) |
| Emotional tone |  |  |  |  |
| Ambiguous | 10 (10%) | 3 (7%) | 1 (17%) | 6 (13%) |
| Positive | 54 (55%) | 25 (54%) | 3 (50%) | 26 (55%) |
| Negative | 26 (26%) | 14 (30%) | 1 (17%) | 11 (23%) |
| Mixed | 9 (9%) | 4 (9%) | 1 (17%) | 4 (9%) |
| Purpose for posting |  |  |  |  |
| Ambiguous | 6 (6%) | 2 (4%) | - | 4 (9%) |
| Educate | 4 (4%) | 2 (4%) | 1 (17%) | 1 (2%) |
| Help others | 5 (5%) | 3 (7%) | - | 2 (4%) |
| Interact | 5 (5%) | 2 (4%) | 1 (17%) | 2 (4%) |
| Seek support | 2 (2%) | - | - | 2 (4%) |
| Share feelings | 35 (35%) | 17 (37%) | - | 18 (38%) |
| Share humorous content | 9 (9%) | 5 (11%) | - | 4 (9%) |
| Share what they were doing | 33 (33%) | 15 (33%) | 4 (67%) | 14 (30%) |
