# Supplementary material for: Visual content and thematic analyses of images shared on social media before and after episodes of self-harm in a UK clinical youth sample
Source: BMJ Open. 2026 Jan 19;16(1):e103456. doi: 10.1136/bmjopen-2025-103456 (PMC12820819; doi:10.1136/bmjopen-2025-103456)
Supplement: online supplemental file 5 [file bmjopen-16-1-s005.docx]

**Supplementary File 5: Description and illustrative examples for themes 1 to 8**

| **Theme** | **Sub-themes** | **Description** | **Example** |
| --- | --- | --- | --- |
| Participation in activities that support wellbeing | Enjoyable activities  Creative outlets  Health-focused actions | The first theme describes images that depicted activities that support wellbeing, typically represented through photographs. This included engagement in pleasant activities, creative outlets and health-focused actions, typically represented in photographs. | An image of a young person playing an electric guitar with their hand on the guitar strings centred, with a caption expressing their passion for playing the instrument. |
| Representations of love and relationships | Social connectedness  Loss of love | The second theme addressed images reflecting love and relationships, comprising images that reflect diverse forms of social connectedness and loss of love. Images were used to convey emotions rather than to seek engagement or evoke a response and were largely personal in nature. | An image comprised of a series of screenshots of an informal online community discussion. |
| Connecting through humour | Light-hearted and trivial content  Humour-based depictions of mental health  Satire  Private jokes | The third theme describes images sharing humorous content, including light-hearted and trivial content, humour-based depictions of mental health, satire and private jokes. Overall, this theme was more often reflected in images with textual content and rarely featuring people compared to other themes. | A textual image about masking mental health difficulties, proposing the individual deserves an “*oscar*" for managing a recent crisis and a positive reference to gender diversity. |
| Expressions of distress and discomfort | Moderate to high levels of distress  Mild upset or frustration | The fourth theme describes images that explicitly communicated distress through text, or visual depictions of discomfort. Moderate to high levels of distress were largely conveyed through textual images, such as a blank background with overlaying text, either generated by other users or themselves. Images portrayed a range of themes including anxiety, depression, hopelessness, interpersonal distress, loneliness, loss, self-harm and suicidality, low self-esteem and social withdrawal. | An image with text on a blank background, using brown tones, declaring that photographs of self-harm wounds “*…should not be shared online…*”. |
| Promoting mental health awareness and support | Raising awareness  Offering support  Receiving support | The fifth theme describes images expressing support and validation of mental health difficulties and this includes advocacy, raising awareness and support. | An image of a male actor with a quote by them overlaying the photograph, describing the harmful portrayal of borderline personality disorder in the media and how this can perpetuate stigmatising attitudes and ignorance within society that can inhibit disclosure. |
| Incongruent atmosphere and tone | Atmosphere  Tone | The sixth theme cuts across several other themes and describes images where there was an incongruence in the atmosphere and the tone. | A selfie photograph of a young person with a neutral or sad facial expression that contrasted with the tone of an applied filter and the caption conveying their enthusiasm. |
| Diverse expressions of self and sexualisation | Individualism  Sexualisation  Blank facial expressions Body positivity | The seventh theme describes images with diverse visual representations of the self and sexualisation and includes celebrating individualism, feminine and masculine sexualisation, blank or neutral facial expressions, and suggestions of body positivity. This theme was predominantly reflected through photographs, less so drawings, with minimal textual content throughout. | A photograph of a young Black female artist featuring a natural hairstyle and masculine tailored suit, in front of a striking purple background. |
| Social activism against injustice | Impersonal  Personal | The eighth theme describes a small number of images posted only on Instagram, depicting activism against different forms of social injustice. | A textual image to communicate a faith-based ruling regarding same-sex marriage, with the addition of a caption that confirmed the personal relevance and expressed their support for those affected. |
