# Supplementary material for: Visual content and thematic analyses of images shared on social media before and after episodes of self-harm in a UK clinical youth sample
Source: BMJ Open. 2026 Jan 19;16(1):e103456. doi: 10.1136/bmjopen-2025-103456 (PMC12820819; doi:10.1136/bmjopen-2025-103456)
Supplement: Supplementary file 6 [file bmjopen-2025-103456.reviewer_comments.pdf]

PEER REVIEW HISTORY

BMJ Open publishes all reviews undertaken for accepted manuscripts. Reviewers are asked to complete a checklist review form and are provided with free text boxes to elaborate on their assessment. These free text comments are reproduced below.

ARTICLE DETAILS

Title (Provisional)

Visual content and thematic analyses of images shared on social media before and after episodes of self-harm in a UK clinical youth sample

Authors

Bye, Amanda; Trevillion, Kylee; Wilson-Lemoine, Emma; Leightley, Daniel; Carter, Ben; Liakata , Maria; Hopper, Jaycee; Dutta, Rina

---

VERSION 1 - REVIEW

**Reviewer**	**1**
**Name**	**Brennan, Cathy**
**Affiliation**	**University of Leeds, Division of Psychological and Social Medicine**
**Date**	**30-May-2025**
**COI**	**None**

Thank you for asking me to review this manuscript that explored the nature and content of images posted on social media around the time of self-harm in young people. This study was part of a larger study of social media and smartphone use and its relationship to self-harm.

This is obviously a timely topic area and the study is linking observable social media activity with individual participant data on self-harm which has huge potential to contribute meaningfully to our understanding of the complex links between social media use and self-harm. I would question to some extent the claim that we can “objectively explore the link” as the authors assert in the introduction – the data used in this study has obvious advantages over self-report activity, not least the ability to link temporally with other events. However, there is still a high level of subjective interpretation necessary in exploring potential links between two associated events.

The manuscript is generally well-written and the authors give a good level of detail on their approach to analysis and the contribution of the research team and PPI to the study. Much thought has been given to an analysis plan and they have undertaken a rigorous analysis of the images selected. However, the contribution of the results to understanding the links

between social media use and self-harm are at present unclear and I think there is some work to do to maximise the contribution of this study to the current debates.

I think there are three significant issues that need addressing.

1. Sample and sampling strategy:

More detail is needed about the participants included in this study in relation to the wider study – it is unclear why only 20 participants were included out of a potential 362. To situate this subsample it would be useful to understand first how many of the 362 had a self-harm event during the study and then how many of these posted anything on social media during their event window. Does this mean only 20 participants had posted images on social media in the time frame of an event? What were the others doing? I need to be able to understand how usual/unusual is it for only 20 participants to have posted an image on social media in a two-week window.

More detail and justification is also needed regarding the social media data sampling strategy. First, what data was actually available – the authors refer to images posted – is this images posted by the participant or might it include images in which they had been tagged and appear on their feed but were posted by others? Second, why sample only images and only selective images? The authors note that if multiple identical images were excluded and not all images posted by participants were included in the analysis – it is not clear why these decisions were made rather than include all images in the analysis.

2. One of the things I am struggling to understand in this manuscript is the purpose of this particular study and how it helps us understand the complex relationship between self-harm and social media use. The authors give the overall aim of the 3S-YP study and state that this paper presents a visual content and thematic analysis of images posted around an event of self-harm. However, it is not clear how this analysis feeds into the overall aim of the 3S-YP study. What is the aim of an analysis of images? There are potentially multiple aims - at present it is not clear what questions the presented analysis is seeking to answer, or if exploratory, what is the purpose of the exploration. Addressing this will help with justifying the sampling as requested above so the reader can make a judgement about the robustness of the data in relation to its purpose.

3. The results also lacked clarity. I struggled to understand what the key messages were from the manuscript. Having a clearer aim for this study could help with this if the results were structured better to address this.

For example, I was unsure what the numbers represented in the posting behaviour and visual content sections. It wasn't clear if the numbers in brackets were about images or about participants. If they were about images, then what is the significance given you haven't analysed all the images? I wasn't sure why it mattered that some participants used particular platforms more, or that most images were photographs. If this study is about the event window then what I want to understand is the nature of an individual participants

posting in this window and how this differs across their own timeline and that of other participants – it was difficult to get this from the results presented.

One key result in the thematic analysis would seem to be that on the event day itself, content seemed to be focused on pleasure/light humour, which was different to pre and post windows that also contained expressions of distress – this reflection seemed to get lost a little in the way the thematic results were presented as themes.

I do apologise to the authors for being negative about the manuscript, there is a lot of potential in the study but at the moment it lacks a clear narrative. There are multiple ways the study could address this depending on what the specific purpose of the manuscript is so I have tried not to be too prescriptive. For example, some of the discussion focused on the discourse around the sharing of harmful images and the relationship to self-harm – some of this worry is about the viewing of images rather than the posting. I think it would strengthen this manuscript to interrogate the dual role of sharing images in more depth and be clearer about what this study contributes to understanding the relationship between images and self-harm. There was some interesting reflections in the discussion about a potential change in online identity curation around a self-harm event and it is here that this manuscript is best placed to contribute – however, this manuscripts' contribution to these discussions could be strengthened significantly if there was more focus in the analysis on how posting activity may (or may not) change around an event window rather than, or in addition to, how it might change within a window.

There were a couple of minor editorial points:

Pg 6, line 6: not consistent in referencing style

There was a lot of use of > to signify amounts, personally I would prefer to read "more than three"/ "three or more" over the use of symbols as this helps the narrative flow – however, this is just a stylistic point so pleased feel free to ignore.

**Reviewer**	**2**
**Name**	**Benau, Erik M**
**Affiliation**	**Department of Psychology, SUNY Old Westbury, Old Westbury, NY, USA.**
**Date**	**13-Oct-2025**
**COI**	**None**

Intro:

The introduction is generally well-written, and the rationale for focusing on social media imagery is clear. However, I was left wondering about how this work fits within the

broader literature on *predicting or identifying self-harm behavior* from online activity. The authors briefly cite prior work on users encouraging self-harm and engaging with such content, but less attention is given to existing predictive or risk-indicator research (e.g., on suicide ideation or attempts). Given that there is a fairly robust literature on social media signals for suicide risk, highlighting whether a similar evidence base exists—or noting its absence—for self-harm specifically would help to position the contribution of this study more clearly

The authors state "we do not yet know enough about the posting behaviours of young people who self-harm" but could sharpen this by distinguishing between the lack of predictive/early warning research on self-harm and the existing descriptive work on suicide and mental health more broadly. Put another way: what is "enough" and "enough to do what?" Why is this important? Clarifying this would help position the study not just as *filling a gap*, but as addressing a specific type of gap (e.g., early detection vs. descriptive understanding) and would give the reader a clearer sense of its potential applied impact. Not all gaps need to be filled, some are more useful than others; what role would this play?

The authors use "self-harm" throughout but could briefly clarify how they are conceptualizing it vs. suicidality up front. The distinction is buried later in Methods, but would be clearer if defined early in the Intro.

The flow from describing prior harms (cyberbullying, exposure to harmful content) to discussing qualitative methods is slightly abrupt. A short transitional sentence framing *why* qualitative image analysis is uniquely useful here would smooth that.

Methods:

The level of detail provided about the research team and coding procedures seems unusually extensive for the main text. While it is appropriate to note who conducted the coding and how reliability and ethical safeguards were ensured—particularly given the sensitive nature of self-harm content—the current description is longer and more granular than is typical. This could likely be condensed to a brief statement in the Methods clarifying the team's role, background, and training, with the full procedural details (e.g., training protocols, support measures, team structure) moved to supplementary materials. Doing so would improve clarity and readability without sacrificing transparency.

Further, while the manuscript notes that trained researchers reviewed and coded posts, it does not specify their background, training, or relevant expertise. Given the sensitivity of self-harm content, it would strengthen the Methods to clarify who conducted the coding (e.g., research assistants, clinicians, lived-experience contributors), what training they received, and how coder wellbeing and consistency were supported. This information is particularly relevant for evaluating the reliability, validity, and ethical oversight of the coding process.

I will note that I don't typically conduct or review qualitative studies, so please correct me if I'm unfamiliar with best practices here.

Unless I'm missing it, it's not clear how "self-harm" is defined in this study. Given how critical this term is, and that the term is used throughout, an explicit operational definition is necessary. Because "self-harm" can encompass a wide range of behaviors—from non-suicidal self-injury to suicide attempts—and can vary substantially between clinical and research contexts, this is an important omission. I strongly encourage the authors to specify how self-harm was defined (e.g., inclusion/exclusion criteria, relevant diagnostic or administrative codes, behavioral thresholds) and how this informed both the sample and the image coding procedures. This is particularly important for interpretability and comparability with prior work.

The study design and timeline are somewhat buried in the procedural detail. It might strengthen the Methods to explicitly state the design (e.g., "retrospective cohort study linking hospital records and Instagram posts") and provide a clear timeline of data collection and analysis at the outset.

The time windows for inclusion of posts relative to self-harm episodes could be described more precisely (e.g., number of days pre- and post-event, handling of multiple events, selection criteria when multiple posts were available). This is especially important given the study's stated focus on chronology.

The selection and exclusion criteria for participants and posts could be clarified. It would be helpful to specify how participants were identified, how missing or insufficient data were handled, and how posts were selected or excluded for coding.

Similarly, the ethics section could provide more procedural detail about consent processes, privacy protections, and safeguards for handling sensitive self-harm imagery. Even a brief clarification here would strengthen the ethical transparency of the study.

Results:

In general, this section is well stated.

The Results section reads primarily as a description of the absence of self-harm imagery rather than as a structured analysis addressing the research aims. If this absence is in itself a key finding, it might be worth framing and contextualizing it more explicitly—e.g., what assumptions or prior narratives this challenges, or what it implies for early-warning or detection efforts.

The Results could be strengthened by presenting the descriptive data more systematically (e.g., clear counts/percentages for posts in each coding category and time window). As written, it's hard to tell whether the absence of self-harm imagery reflects a meaningful finding or simply sample and analytic constraints. If counts are not important or part of this approach to coding, then I would say make that clearer in the methods section for more novice readers like me who may not know that.

The results would also benefit from richer illustrative content (e.g., representative image descriptions or themes), even if self-harm imagery was rare. This would give readers a clearer sense of what kinds of content *were* present.

Discussion:

The authors state that filtering of online posts was a likely source of reduced The Discussion would be strengthened by explicitly situating these findings in the context of improved automated moderation and filtering on social media platforms. The low prevalence of explicit self-harm imagery is likely not just a sample artifact but reflects a changing digital landscape. This makes studies like the present one particularly relevant, as future detection efforts will need to focus on more subtle or indirect indicators rather than overt content.

The increased prevalence of “self-care and wellness” content (among others) could be more clearly framed as a meaningful result. Even if not a strong candidate for intervention on its own, it may represent one of a set of weaker, indirect indicators that—combined with other digital behavioral data—could inform future risk detection work.

The limitations section could be strengthened by explicitly acknowledging the platform-specific context of these findings and the likelihood that moderation algorithms contributed to the low prevalence of self-harm imagery. This would help clarify generalizability and highlight why indirect content categories require cautious interpretation if used for intervention.

The authors could make a stronger forward-looking statement connecting these findings to practical detection and prevention strategies. For example, they might emphasize how subtle, non-harm content could be integrated into broader multimodal models (e.g., language, network structure) rather than functioning as a standalone flag. This would underscore the applied relevance of their work despite the low prevalence of explicit imagery.

Small notes:

Abstract: either include all percentages or don’t include them

A few sentences, particularly in the Introduction and Discussion, could be broken into shorter units for clarity and flow.

VERSION 1 - AUTHOR RESPONSE

**Reviewer 1 comments**	**Author response**
Thank you for asking me to review this manuscript that explored the nature and content of images posted on social media around the time of self-harm in young people. This study was part of a larger study of social media and smartphone use and its relationship to self-harm.<br><br>This is obviously a timely topic area and the study is linking observable social media activity with individual participant data on self-harm which has huge potential to contribute meaningfully to our understanding of the complex links	We would like to thank Reviewer 1 for their time and effort reviewing our revised manuscript and for all their positive and constructive comments, which have enabled us to improve our paper.

between social media use and self-harm. I would question to some extent the claim that we can "objectively explore the link" as the authors assert in the introduction – the data used in this study has obvious advantages over self-report activity, not least the ability to link temporally with other events. However, there is still a high level of subjective interpretation necessary in exploring potential links between two associated events.<br><br>The manuscript is generally well-written and the authors give a good level of detail on their approach to analysis and the contribution of the research team and PPI to the study. Much thought has been given to an analysis plan and they have undertaken a rigorous analysis of the images selected.<br><br>I think there are three significant issues that need addressing.	Thank you for highlighting this point about objectivity. We have revised the relevant section in the introduction accordingly (see page 7 - paragraph 2 - line 1-2).
1. Sample and sampling strategy:<br><br>More detail is needed about the participants included in this study in relation to the wider study – it is unclear why only 20 participants were included out of a potential 362. To situate this subsample it would be useful to understand first how many of the 362 had a self-harm event during the study and then how many of these posted anything on social media during their event window. Does this mean only 20 participants had posted images on social media in the time frame of an event? What were the others doing? I need to be able to understand how usual/unusual is it for only 20 participants to have posted an image on social media in a two-week window.<br><br>More detail and justification is also needed regarding the social media data sampling strategy. First, what data was actually available – the authors refer to images posted – is this images posted by the participant or might it include images in which they had been tagged and appear on their feed but were posted by others? Second, why sample only images and only selective images? The authors note that if multiple identical images were excluded and not all images posted by participants were included in the analysis – it is not clear why these decisions were made rather than include all images in the analysis.	Thank you for raising these questions about the sampling strategy.<br><br>- Of the total sample, 122 had at least one self-harm event. Among these, 25 had social media posts within the event window, 20 of whom had image data available. We have included this detail under a new sub-heading – *Sampling strategy* – to help situate the subsample within the wider study (see page 14-15).<br><br>- Only images posted by the user are available in their data provided by the social media platforms, and not images in which the user has been tagged by others. We have clarified this restriction in the methods under the *Social media data* sub-heading (see page 13).<br><br>- This analysis focused on images posted by participants on social media, for the reasons now outlined in the introduction. Images also represented the main source of qualitative data available within the defined event window, and including textual posts would have required a different analytical approach.<br><br>- The rationale for removing duplicate images and limiting the number of images from a single participant within the same day, time slot and modality was an a priori decision to improve efficiency and reduce potential data bias. We have moved the information about data selection to the new sub-section – *Sampling strategy* - and provided further details where necessary to ensure clarity (see page 14-15). We have also acknowledged the potential implications in the discussion (see page 35 - paragraph 1 - line 9-10).
2. One of the things I am struggling to understand in this manuscript is the purpose of this particular study and how it	This analysis was exploratory, aimed at improving understanding of online self-expression during periods of self-

helps us understand the complex relationship between self-harm and social media use. The authors give the overall aim of the 3S-YP study and state that this paper presents a visual content and thematic analysis of images posted around an event of self-harm. However, it is not clear how this analysis feeds into the overall aim of the 3S-YP study. What is the aim of an analysis of images? There are potentially multiple aims - at present it is not clear what questions the presented analysis is seeking to answer, or if exploratory, what is the purpose of the exploration. Addressing this will help with justifying the sampling as requested above so the reader can make a judgement about the robustness of the data in relation to its purpose.	harm, while also attempting to identify subtle changes that may signal early indicators of risk. We consider that findings such as these may inform future research, and thinking about the types of support young people might find helpful. We have revised the aims in the abstract and introduction and clarified the purpose and value of this research in the introduction to make these points explicit (see page 4 and pages 8 - paragraph 2 to page 9 paragraph 1).
3. The results also lacked clarity. I struggled to understand what the key messages were from the manuscript. Having a clearer aim for this study could help with this if the results were structured better to address this. For example, I was unsure what the numbers represented in the posting behaviour and visual content sections. It wasn't clear if the numbers in brackets were about images or about participants. If they were about images, then what is the significance given you haven't analysed all the images? I wasn't sure why it mattered that some participants used particular platforms more, or that most images were photographs.	- As noted above, we have revised the manuscript throughout to ensure greater clarity regarding the aims, insights generated and potential implications of these.<br>- The posting patterns and visual content findings are intended to offer some useful context and visual characterisation of images shared on social media during periods of self-harm. Findings such as these could inform computational image analysis approaches that involve extracting visual and textual features from images. We have revised the relevant sub-sections so that numbers are reported at the image level rather than the participant level, improving readability and ensuring consistency with the other results presented (see pages 20-22).<br>- We have also added two tables with the visual content and thematic findings as supplementary information.
If this study is about the event window then what I want to understand is the nature of an individual participants posting in this window and how this differs across their own timeline and that of other participants – it was difficult to get this from the results presented.	We agree that analysing the image data at the individual level could generate some interesting insights. We intend to use the present analysis as a foundation for this work, and a subsequent paper will be created which presents individual level data, as noted in the strengths sub-section (see page 34 - paragraph 2 - line 7-9).
One key result in the thematic analysis would seem to be that on the event day itself, content seemed to be focused on pleasure/light humour, which was different to pre and post windows that also contained expressions of distress – this reflection seemed to get lost a little in the way the thematic results were presented as themes.	We have grouped all temporal thematic changes under a new sub-section so that the subtle changes we identified are more accessible to the reader (see page 29-30).
There is a lot of potential in the study but at the moment it lacks a clear narrative. There are multiple ways the study could address this depending on what the specific purpose of the manuscript is so I have tried not to be too prescriptive. For example, some of the discussion focused on the discourse around the sharing of harmful images and the relationship to	We have revised sections of the Discussion to present a clear narrative:<br>The following sentence in the discussion to acknowledge concern about both sharing and viewing – 'While much of the current discourse is framed around the harmful effects of sharing and viewing graphic self-harm images online, our

self-harm – some of this worry is about the viewing of images rather than the posting. I think it would strengthen this manuscript to interrogate the dual role of sharing images in more depth and be clearer about what this study contributes to understanding the relationship between images and self-harm.	results suggest that sharing such images may not be widespread, at least on platforms with greater public visibility' (see page 31 - paragraph 1 - line 2-4).<br>- We have added the following sentence to the discussion regarding the dual purpose of image sharing - 'Among the few images referencing self-harm in the textual content, both personal and socially driven motivations for sharing were reflected. Content seemed to serve as a means of emotional expression and seeking or offering support – this pattern was also observed in the other mental health-related images.' (see page 31 - paragraph 1 - line 12-15).
There was some interesting reflections in the discussion about a potential change in online identity curation around a self-harm event and it is here that this manuscript is best placed to contribute – however, this manuscripts' contribution to these discussions could be strengthened significantly if there was more focus in the analysis on how posting activity may (or may not) change around an event window rather than, or in addition to, how it might change within a window.	Thank you for this feedback. As noted above, we have revised the results, including grouping all temporal thematic changes under a new sub-section – *Temporal thematic changes* (pages 29-30). We have also reviewed and revised the entire discussion section to ensure the identified temporal changes are highlighted.
There were a couple of minor editorial points:<br>Pg 6, line 6: not consistent in referencing style	This referencing error has now been resolved.
There was a lot of use of > to signify amounts, personally I would prefer to read "more than three"/ "three or more" over the use of symbols as this helps the narrative flow – however, this is just a stylistic point so pleased feel free to ignore.	We have revised the manuscript accordingly to assist with the narrative.
**Reviewer 2 comments**	**Author response**
Intro:<br>The introduction is generally well-written, and the rationale for focusing on social media imagery is clear. However, I was left wondering about how this work fits within the broader literature on predicting or identifying self-harm behavior from online activity. The authors briefly cite prior work on users encouraging self-harm and engaging with such content, but less attention is given to existing predictive or risk-indicator research (e.g., on suicide ideation or attempts). Given that there is a fairly robust literature on social media signals for suicide risk, highlighting whether a similar evidence base exists—or noting its absence—for self-harm	We would like to thank Reviewer 2 for their time and effort reviewing our manuscript and for all their constructive comments enabling us to improve upon our original paper.<br><br>Thank you for this suggestion about framing the relevance of this research. Whilst still of value, existing evidence (including big data analytics) lacks individual-level and contextual data. This limits the ability to infer risk and apply the findings at an individual level. This exploratory analysis, using a novel data linkage, has the potential to address these gaps and provide insights into online self-expression and possible early indicators of risk. Such findings could inform statistical and computational analytical approaches, and prevention and

specifically would help to position the contribution of this study more clearly<br><br>The authors state "we do not yet know enough about the posting behaviours of young people who self-harm" but could sharpen this by distinguishing between the lack of predictive/early warning research on self-harm and the existing descriptive work on suicide and mental health more broadly. Put another way: what is "enough" and "enough to do what?" Why is this important? Clarifying this would help position the study not just as filling a gap, but as addressing a specific type of gap (e.g., early detection vs. descriptive understanding) and would give the reader a clearer sense of its potential applied impact. Not all gaps need to be filled, some are more useful than others; what role would this play?	intervention strategies. We have added further information about the rationale / value of this research to the introduction (see page 9 - paragraph 1).
The authors use "self-harm" throughout but could briefly clarify how they are conceptualizing it vs. suicidality up front. The distinction is buried later in Methods, but would be clearer if defined early in the Intro.	We consider self-harm to encompass a broad range of behaviours, including suicide attempts. To clarify this upfront, we have added the UK National Institute for Health and Care Excellence (NICE) definition for self-harm to the introduction (see page 7 - paragraph 1 - line 1-2).<br><br>Further detail on the self-harm definition used in the 3S-YP study has been added to the methods section under *Measures – Self-harm* sub-heading, as this detail would have been too extensive for the introduction (see pages 12-13).
The flow from describing prior harms (cyberbullying, exposure to harmful content) to discussing qualitative methods is slightly abrupt. A short transitional sentence framing why qualitative image analysis is uniquely useful here would smooth that.	We have revised this section to improve the flow (see page 7).
Methods:<br><br>The level of detail provided about the research team and coding procedures seems unusually extensive for the main text. While it is appropriate to note who conducted the coding and how reliability and ethical safeguards were ensured—particularly given the sensitive nature of selfharm content—the current description is longer and more granular than is typical. This could likely be condensed to a brief statement in the Methods clarifying the team's role, background, and training, with the full procedural details (e.g., training protocols, support measures, team structure) moved to supplementary materials. Doing so would improve clarity and readability without sacrificing transparency.	We have revised the *Research team* section to remove details captured elsewhere in the manuscript while maintaining transparency, in line with best practice for reporting qualitative research (see pages 10-11).
Further, while the manuscript notes that trained researchers reviewed and coded posts, it does not specify their background, training, or relevant expertise. Given the	Researcher training and expertise, along with analysis and clinical supervision arrangements, were in accordance with best practice for conducting qualitative analysis and are

sensitivity of self-harm content, it would strengthen the Methods to clarify who conducted the coding (e.g., research assistants, clinicians, lived-experience contributors), what training they received, and how coder wellbeing and consistency were supported. This information is particularly relevant for evaluating the reliability, validity, and ethical oversight of the coding process.<br><br>I will note that I don't typically conduct or review qualitative studies, so please correct me if I'm unfamiliar with best practices here.	detailed under the *Research Team* and *Analysis* subheadings (see page 10-11 and pages 15-16).
Unless I'm missing it, it's not clear how "self-harm" is defined in this study. Given how critical this term is, and that the term is used throughout, an explicit operational definition is necessary. Because "self-harm" can encompass a wide range of behaviors—from non-suicidal self-injury to suicide attempts—and can vary substantially between clinical and research contexts, this is an important omission. I strongly encourage the authors to specify how self-harm was defined (e.g., inclusion/exclusion criteria, relevant diagnostic or administrative codes, behavioral thresholds) and how this informed both the sample and the image coding procedures. This is particularly important for interpretability and comparability with prior work.	Further detail on the self-harm definition used in the 3S-YP study has been added to the *Methods* section under *Measures – Self-harm* (see pages 12-13). In the *Study design* sub-section (page 9), readers are also directed to the baseline cohort paper for full methods details.
The study design and timeline are somewhat buried in the procedural detail. It might strengthen the Methods to explicitly state the design (e.g., "retrospective cohort study linking hospital records and Instagram posts") and provide a clear timeline of data collection and analysis at the outset.	We have revised the description of the study design in the *Study design* sub-section (page 9), to make clear the prospective nature and novel data linkages. We have also added further information about the timeline to the *Recruitment and data collection* sub-section (see page 11 - paragraph 2).
The time windows for inclusion of posts relative to self-harm episodes could be described more precisely (e.g., number of days pre- and post-event, handling of multiple events, selection criteria when multiple posts were available). This is especially important given the study's stated focus on chronology.	The event window is defined in the Study design sub-section as 'the seven days before an event, the day of the event itself and the seven days following an event – herein termed the 'event window''. To ensure clarity, details about data selection are now presented under a new sub-heading – *Sampling strategy* (see page 14-15).
The selection and exclusion criteria for participants and posts could be clarified. It would be helpful to specify how participants were identified, how missing or insufficient data were handled, and how posts were selected or excluded for coding.	As above, we have moved the information about data selection to a new sub-section – *Sampling* strategy – and provided further details where necessary to ensure clarity.
Similarly, the ethics section could provide more procedural detail about consent processes, privacy protections, and safeguards for handling sensitive self-harm imagery. Even a	Further information about these ethical aspects have been added to the Recruitment and data collection, and Ethics approval sub-sections (see from page 11 - paragraph 2 - line 10 to page 12 - paragraph 2 - line 6).

brief clarification here would strengthen the ethical transparency of the study.	
Results:<br>In general, this section is well stated.<br>The Results section reads primarily as a description of the absence of self-harm imagery rather than as a structured analysis addressing the research aims. If this absence is in itself a key finding, it might be worth framing and contextualizing it more explicitly—e.g., what assumptions or prior narratives this challenges, or what it implies for early-warning or detection efforts.	The exploratory nature of this analysis has now been made explicit in the introduction so we anticipate that this will help frame and contextualise this work (see from page 8 - paragraph 2 to page 9 - paragraph 1). The absence of self-harm imagery is an interesting finding, but the analysis and results are intended to reflect online self-expression through imagery more broadly. We have revised the result section to improve the flow and ensure key findings are more clearly presented.
The Results could be strengthened by presenting the descriptive data more systematically (e.g., clear counts/percentages for posts in each coding category and time window). As written, it's hard to tell whether the absence of self-harm imagery reflects a meaningful finding or simply sample and analytic constraints. If counts are not important or part of this approach to coding, then I would say make that clearer in the methods section for more novice readers like me who may not know that.	We have revised the descriptive findings to ensure consistency. The following sentence has been added to the *Analysis* section to outline the purpose of the image counts – 'Image counts were then prepared to show how many images displayed each visual feature' (see page 16 - paragraph 1 - line 3). We have also added a table with the visual content findings as supplementary information (see Supplementary File 3).
The results would also benefit from richer illustrative content (e.g., representative image descriptions or themes), even if self-harm imagery was rare. This would give readers a clearer sense of what kinds of content were present.	We have reviewed and revised the *Results* section where appropriate, whilst ensuring that participant data privacy remains fully safeguarded.
Discussion:<br>Discussion would be strengthened by explicitly situating these findings in the context of improved automated moderation and filtering on social media platforms. The low prevalence of explicit self-harm imagery is likely not just a sample artifact but reflects a changing digital landscape. This makes studies like the present one particularly relevant, as future detection efforts will need to focus on more subtle or indirect indicators rather than overt content.	Thank you very much for this feedback, we have included this in the *Discussion* section (see page 31 - paragraph 1 - lines 6-12).
The increased prevalence of "self-care and wellness" content (among others) could be more clearly framed as a meaningful result. Even if not a strong candidate for intervention on its own, it may represent one of a set of weaker, indirect indicators that—combined with other digital behavioral data—could inform future risk detection work.	Thank you very much for this helpful comment - we have included this in the revised *Discussion* (see page 32 – paragraph 1 – lines 5-8).
The limitations section could be strengthened by explicitly acknowledging the platform specific context of these findings and the likelihood that moderation algorithms contributed to	We have included the following sentence in the *Strengths and limitations* section regarding the generalisability of the findings – '*Although the platforms examined in this analysis*

the low prevalence of self-harm imagery. This would help clarify generalizability and highlight why indirect content categories require cautious interpretation if used for intervention.	*are widely used by young people, our findings may not generalise to other platforms, particularly those offering greater anonymity and looser moderation policies*' (see from page 34 – paragraph 3 – line 1 to page 35 – paragraph 1 – line 1).
The authors could make a stronger forward-looking statement connecting these findings to practical detection and prevention strategies. For example, they might emphasize how subtle, non-harm content could be integrated into broader multimodal models (e.g., language, network structure) rather than functioning as a standalone flag. This would underscore the applied relevance of their work despite the low prevalence of explicit imagery.	Thank you very much for this recommendation. We have outlined some potential future avenues for future research to the discussion section and provided a forward-looking statement in the Conclusion (see page 36 – paragraph 1 – lines 1-8).
Small notes:<br>Abstract: either include all percentages or don't include them	We have chosen to omit the percentages which were superfluous and revised the abstract accordingly.
A few sentences, particularly in the Introduction and Discussion, could be broken into shorter units for clarity and flow.	We have revised the introduction and discussion accordingly.

VERSION 2 - REVIEW

Reviewer 1
Name Brennan, Cathy
Affiliation University of Leeds, Division of Psychological and Social Medicine
Date 19-Dec-2025
COI

Thank you for asking me to review this resubmission of a manuscript that explored the nature and content of images posted on social media around the time of self-harm in young people. The authors have addressed all my initial concerns and I think the manuscript now has a clear narrative that offers an important contribution to the evidence on social medial use and its relationship with self-harm. Appropriate attention and acknowledgement has been made to the limitations of the study. I look forward to hearing more results from the 3S-YP study.
